# Fermentation of House Crickets (*Acheta domesticus*): Boosting Quality and Functionality in Cricket-Based Food Ingredients

**DOI:** 10.3390/foods14234003

**Published:** 2025-11-22

**Authors:** Seyed Mohammad Hasan Haghayeghi, Andrea Osimani, Lucia Aquilanti

**Affiliations:** Dipartimento di Scienze Agrarie, Alimentari ed Ambientali (D3A), Università Politecnica delle Marche, Via Brecce Bianche, 60131 Ancona, Italy; s.m.h.haghayeghi@pm.univpm.it (S.M.H.H.); l.aquilanti@univpm.it (L.A.)

**Keywords:** edible insects, fermentation, cricket powder, functional food, lactic acid bacteria, protein hydrolysates, consumer acceptance, food innovation

## Abstract

This review examines the nutritional and functional potential of *Acheta domesticus*, the impact of fermentation on its biochemical and microbiological properties, and its application in food ingredients and products. Relevant literature was reviewed on the composition, fermentation behavior, product development, and consumer perceptions related to cricket-based ingredients, with a focus on fermented applications and microbiota interaction. Fermentation improves the safety, digestibility, flavor, and nutritional value of cricket powder. Lactic acid bacteria (e.g., *Lactiplantibacillus plantarum* and *Latilactobacillus curvatus*) enhanced substrate acidification, reduced biogenic amines and acrylamide levels, and contributed to desirable volatile compounds production. Additionally, fermentation using yeasts like *Yarrowia lipolytica* and *Debaryomyces hansenii* resulted in the production of antimicrobial substances, reduction in chitin, and an increase in the matrix digestibility. Fermented cricket-based ingredients have been successfully applied to bread, biscuits, yogurt, and beverages. Protein hydrolysates produced by fermentation exhibited antioxidant, anti-aging, and preservative properties, expanding potential beyond food. Consumer acceptance was highest when insects were integrated into familiar and visually unobtrusive food formats. To conclude, *A. domesticus* shows great promise as a sustainable and functional food ingredient. Fermentation offers a key strategy to overcome safety, sensory, and acceptability barriers.

## 1. Introduction

As the global population continues to rise and climate change intensifies, the need for sustainable, nutrient-dense, and resource-efficient food sources is becoming increasingly urgent. Edible insects, particularly *Acheta domesticus* (house cricket), have gained attention as a promising alternative protein source due to their rich nutritional composition, high feed conversion efficiency, and low environmental impact [1]. Crickets provide a complete amino acid profile, essential fatty acids, vitamins, and minerals, making them attractive for both food security and functional nutrition [1,2]. Despite these advantages, consumer acceptance of insect-based foods remains limited in many regions, especially in Western cultures, where psychological and cultural barriers such as disgust and food neophobia often prevail. Moreover, challenges related to their safety, especially considering microbial contamination, potential allergenicity, and sensory quality, must be addressed to enable the successful integration of cricket-based ingredients into mainstream food products [3,4].

In the European Union (EU), the commercialization of edible insects is regulated under the Novel Food Regulation (EU) 2015/2283. Before entering the market, insect-based products must receive authorization, which relies on a safety evaluation conducted by the European Food Safety Authority (EFSA). Manufacturers are required to submit applications to the European Commission, and approvals are granted through Commission Implementing Regulations. Regarding crickets, in 2022, the Commission Implementing Regulation (EU) 2022/188 authorized the placing on the market of frozen, dried and powdered forms of *A. domesticus* as a novel food. Moreover, in June 2024, the EFSA issued the scientific opinion on the Safety of *A. domesticus* powder as a Novel food pursuant to Regulation (EU) 2015/2283 followed by the scientific opinion on safety of frozen, dried and powder forms of house crickets (*A. domesticus*) as a novel food pursuant.

In addition, insect-derived foods must comply with labeling rules, as well as general standards for food hygiene, traceability, storage, and transportation. As a result, farming and marketing crickets as food for humans is now a tangible reality in the EU. This advancement nonetheless presents various technological hurdles, including the need to refine rearing systems, guarantee consistent safety and quality standards, and design processing techniques that both preserve nutritional benefits and meet consumer expectations.

Crickets are commercially available either as whole insects or in the form of powder or paste. The paste is generally produced by insect blanching and grinding, while the powder is typically obtained by insect blanching, lyophilization, and grinding.

Fermentation has emerged as a promising biotechnological approach to address many of the challenges associated with the use of insect-based ingredients [2,4]. Through microbial and enzymatic processes, fermentation not only enhances the nutritional and functional properties of insects but also improves their sensory characteristics and microbiological safety. Of note, the obtained enhancements depend on the matrix and the microorganism used.

Fermentation and microbial bioprocessing have also been recommended as powerful tools to enhance nutritional and environmental performance of alternative protein systems, including edible insects. Some studies have demonstrated that fermentation of edible insects such as *Tenebrio molitor*, *A. domesticus*, and *Locusta migratoria* using *Lactobacillus plantarum* or mixed starter cultures can change amino acid profiles, improve mineral bioavailability, and modulate oxidative stability [5]. Beside compositional and sensory enhancements, co-fermentation of microorganisms and edible insects also offers circular bioeconomic advantages. For instance, probiotic lactic acid bacteria (LAB) have been shown to degrade pesticide residues and modulate microbiota composition, in addition to the sensory enhancement and safety of fermented foods [6]. Furthermore, combining insect rearing with microbial pre-treatments has been reported to improve substrate utilization and nutrient conversion efficiency [7]. At the systems level, the integration of these technologies is in accordance with the Circular Bioeconomy (CBE) framework [8,9], which emphasizes (1) efficient resource use through valorization of biowastes (e.g., insect feed, algae, fungal biomass), (2) water conservation, (3) energy recovery, (4) greenhouse gas reduction, and (5) improved soil health. Collectively, these developments illustrate how microbial fermentation, nutritional innovation, waste minimization, and sustainability can jointly support the next generation of food systems.

To the authors’ knowledge, a few literature reviews dealing with fermentation of insects have been published [10,11,12,13]. However, no reviews exclusively focused on the fermentation of *A. domesticus* are available in the scientific literature.

This review advances current knowledge by shedding light on a relatively underexplored area: the role of fermentation in optimizing the nutritional, sensory, and functional qualities of *A. domesticus*. By integrating scientific insights with potential industrial applications, this review aims to support the development of novel food ingredients and products derived from this edible insect.

## 2. Why *A. domesticus*?

*A. domesticus* commonly known as house cricket, is increasingly recognized as a nutrient-dense food source with the potential to address global food security and nutritional challenges. Its biochemical profile reveals a well-balanced composition of macronutrients and micronutrients, along with bioactive compounds potentially conferring health benefits.

### 2.1. Protein Content

Regarding protein content, *A. domesticus* typically contains 21.5–75% protein on a dry weight basis, depending on rearing conditions, developmental stage, and processing methods [14,15,16,17,18,19,20,21,22,23]. The protein fraction of *A. domesticus* is characterized by a well-balanced amino acid profile, including all nine essential amino acids. Notably, levels of lysine, leucine, and valine are comparable to or exceed those found in traditional animal protein sources such as beef and poultry [17,21,24]. Non-essential amino acids such as glutamic acid and aspartic acid are also abundant [17,21,24], contributing to umami flavor and potential functional roles in metabolism and gut health [17]. Moreover, the protein hydrolysates derived from cricket protein could be susceptible to the Maillard reaction according to the release of amino acids and peptides during enzymolysis, which may lead to flavor changes in the final cricket protein product [24]. Interestingly, Li et al. [25] have investigated the effects of combining cricket powder and silkworm pupae powder with traditional rice noodles to enhance their nutritional value. The addition of these two powders increased protein content (from 4.96% to 12.06–25.85%) and in-vitro protein digestibility (from 63.25% to 71.61–90.58%).

### 2.2. Fatty Acid Profile

The lipid content of *A. domesticus* ranges from 10–29% (dry weight), and its fatty acid profile includes both saturated and unsaturated fats [15,17,21,24]. Among the fatty acids, palmitic acid (C16:0), oleic acid (C18:1) and linoleic acid (C18:2, an essential omega-6 fatty acid) are predominant (up to 70%) [15,26]. Although levels of omega-3 fatty acids such as α-linolenic acid (C18:3) are generally low, the overall lipid composition contributes favorably to the nutritional quality of the insect [26]. The presence of tocopherols, phospholipids, and sterols, including cholesterol, has also been reported [26].

*A. domesticus* contains a distinct fatty acid profile compared to traditional protein sources. Its fatty acid composition is often like that of poultry, fish, and pork, but it tends to be richer in unsaturated fatty acids, particularly polyunsaturated fatty acids, which constitute 29–31% of the total fatty acids [1,27,28].

### 2.3. Potential Bioactive Compounds (Vitamins, Antioxidants, and Protein Hydrolysates)

*A. domesticus* is a valuable source of several B vitamins, particularly thiamine (B1), riboflavin (B2), niacin (B3), pantothenic acid (B5), pyridoxine (B6), biotin (B7), folic acid (B9), and cobalamin (B12), this latter typically lacking in plant-based diets [1]. It also contains moderate levels of vitamin A (as provitamin A carotenoids), vitamin E (α-tocopherol), vitamin D, and vitamin K [1,21,23,29]. The concentrations of these vitamins not only rival but often exceed those found in traditional meat sources [1], and, in some cases, can fully meet or surpass the recommended daily intakes for both adults and children [1].

Antioxidants are important in minimizing the effects of oxidative stress which are correlated to numerous chronic diseases such as cardiovascular conditions, diabetes, and inflammatory disorders. Recent studies suggest that crickets contain various antioxidant compounds, including polyphenols, peptides, and vitamins with radical-scavenging activity [13,30,31,32]. Chitosan, a valuable biopolymer derived from chitin through deacetylation process and present in *A. domesticus*, has exhibited Reactive oxygen species (ROS)-reducing, antidiabetic, antihyperlipidemic, and antiobesity properties [31,33]. *A. domesticus* has demonstrated antioxidant activity measured to be five times higher than that of fresh orange juice. Additionally, extracts from *A. domesticus* were able to inhibit pancreatic lipase. Ethanolic extracts of *A. domesticus* have demonstrated approximately 80% inhibition in DPPH (2,2-diphenyl-1-picrylhydrazyl) assays which is directly correlated with their phenolic content [34]. Dietary supplementation with cricket powder has been reported to modify inflammatory markers in humans. For example, consuming a breakfast enriched with 25 g of cricket powder reduced tumor necrosis factor (TNF-α) levels in adult humans [13]. Additionally, protein hydrolysates derived from *A. domesticus* have demonstrated in vitro antioxidant capacity, indicating a potential role in reducing oxidative stress and inflammation [29]. However, further research is required to identify specific compounds and validate their bioavailability and efficacy in vivo.

### 2.4. Mineral Content

The total mineral content, often described as ash content, of *A. domesticus* usually ranges from 1.3% to 8.5% on a dry matter [35]. The mineral profile of *A. domesticus* includes macro-minerals such as phosphorus, potassium, calcium, sodium, and magnesium, and critical trace minerals including iron, zinc, manganese, and copper [15,16,21,23,24,27,36,37,38]. Quantitative comparisons reveal that *A. domesticus* contains higher levels of certain minerals such as iron and calcium than conventional meat sources like beef, chicken, and pork [1]. Bioavailability can be influenced by the presence of antinutritional factors such as phytates and chitin; for instance, zinc from insects is well-absorbable by human and iron absorption can be limited by factors like chitin [39], although fermentation and enzymatic treatments have been shown to enhance mineral absorption [31].

### 2.5. Carbohydrate and Fiber Content

*A. domesticus* is a great candidate for low-carbohydrate formulations due to its low carbohydrate content (1.2–4.57%). Crickets usually store carbohydrates in the form of glycogen in their fat body [15,17,19,24,28]. The fiber content has been reported to be around 3.5–19.18%. The most abundant dietary fiber found in *A. domesticus* is a complex carbohydrate called chitin. Chitin has also been supposed to positively influence gut health by promoting the growth of beneficial gut bacteria. It also increases stool bulk and softness, aiding regular bowel movements and potentially preventing conditions like constipation and hemorrhoids. Moreover, it is important to note that fiber content can be strongly influenced by the protein extraction method used [15,17,19,21,24,33,38,40].

### 2.6. Techno-Functional Properties

Beyond its nutritional value, *A. domesticus* exhibits a range of techno-functional properties that make it a promising candidate for incorporation into various food matrices. These functional attributes are crucial for determining the feasibility of using insect-derived ingredients in both conventional and novel food systems. One of the most notable technological advantages of *A. domesticus* powder is its high water-holding capacity (WHC). This characteristic enhances its applicability in aqueous food formulations, where moisture retention is critical. In particular, the high WHC contributes to improved texture, shelf life, and mouthfeel in products such as baked goods, where moisture loss is typically a major challenge [18,41,42,43,44,45].

By retaining water, cricket powder can also influence bread dough rheology and crumb softness, potentially reducing the need for additional hydrocolloids or emulsifiers. Protein extracts from *A. domesticus* have also demonstrated excellent foaming and emulsifying properties, which are essential for the stability and structure of aerated and emulsified food systems such as mousses, whipped toppings, dressings, and meat analogs [18,41]. The foaming capacity is primarily attributed to the amphiphilic nature of certain cricket proteins that can migrate to air–water interfaces, stabilizing bubbles and enhancing texture [18,41]. Similarly, the emulsifying ability of cricket proteins facilitates oil–water dispersion, contributing to the stability and uniformity of emulsions. These properties suggest a potential role for cricket proteins as functional alternatives to egg proteins, soy lecithin, and dairy-derived emulsifiers in certain formulations [41,42,44]. However, the absence of gelation capacity in *A. domesticus* proteins limits their application in products that require heat-set or cold-set gels for structure and firmness, such as puddings, jellies, or meat analogues that mimic whole-muscle texture. This lack of gelation indicates that while cricket proteins are suitable for enhancing texture and stability, they may require blending with other gelling agents (e.g., gelatin, carrageenan, or starches) when used in structural formulations.

### 2.7. Anti-Nutritional Factors and Allergens

The growing interest in *A. domesticus* as a sustainable nutrient-rich source for human consumption has also raised questions regarding its safety, particularly in relation to antinutritional factors and potential allergens. Like many plant- and animal-derived food sources, insects may contain compounds that interfere with nutrient absorption or elicit adverse immune responses. Several studies have identified the presence of antinutritional compounds in *A. domesticus*, including chitin, phytates and tannins [2]. Chitin, a structural polysaccharide found in insect exoskeleton, may reduce protein digestibility and interfere with the bioavailability of micronutrients such as zinc and iron [16,23,46,47]. However, chitin also exhibits potential prebiotic and immunomodulatory properties, suggesting that its impact may vary depending on processing methods and individual health status. Phytates and tannins, though present at lower concentrations compared to plant-based foods, can similarly impair mineral absorption, albeit to a lesser extent [2].

Allergenic risks associated with *A. domesticus* consumption are primarily linked to proteins such as tropomyosin, arginine kinase, and hexamerin, which are known pan-allergens also found in crustaceans and dust mites [16,22,47,48]. Consequently, individuals with shellfish allergies may be at risk of cross-reactive allergic responses upon consuming cricket-based products. Research has indicated that *A. domesticus* tropomyosin remains immunoreactive even after simulated digestion. Furthermore, a cross-reactivity between proteins related to house dust mites (HDM) and cricket proteins has been reported [16,47]. Sensitization and allergic reactions have been documented, particularly in occupational settings or among frequent consumers [47]. Notably, food processing techniques including thermal treatment and fermentation may change protein structures and reduce allergenicity, although further research is needed to establish standardized protocols and assess long-term safety.

## 3. Literature Search Strategy

This review is presented as a narrative overview with scoping elements according to its emerging and interdisciplinary nature of the research objective. This approach was selected to highlight the nutritional, functional, sensory, and technological outcomes of *A. domesticus* powder fermentation for food applications.

The PRISMA 2020 (Preferred Reporting Items for Systematic Reviews and Meta-Analyses) framework [49] was employed to ensure a transparent and structured process of identifying, selecting, and synthesizing literature relevant to the fermentation of *A. domesticus* powder in food applications. Although this paper is not a systematic review or scoping review, PRISMA was utilized to ensure methodological validity and transparency in reporting.

### 3.1. Database Selection and Search Strategy

A structured literature search of published articles on the fermentation of *A. domesticus* powder was performed in three academic databases: Scopus, Web of Science, and PubMed databases on 15 March 2025. The following Boolean search query was applied:

(“Cricket” OR “Acheta” OR “Acheta domesticus” OR “Cricket powder” OR “Cricket flour”) AND (“Fermentation”).

According to the small body of literature in this emerging field, the three selected databases were considered sufficient to retrieve the peer-reviewed studies.

### 3.2. Inclusion and Exclusion Criteria

Articles were selected according to the following inclusion criteria:

(i) Publication in a peer-reviewed journal;
(ii) Research related to the use of house cricket powder in combination with fermentation for the development of food products;
(iii) Written and published in English;
(iv) Articles published between 2018 and 2025.

No restrictions on publication were applied but duplicate reports and documents such as conference proceedings, project reports, theses, and articles unrelated to the application of *A. domesticus* in fermented food systems were excluded.

### 3.3. Data Screening and Management

To manage and screen the literature, Zotero software (version 7.0) [50] was used for reference organization, while Rayyan (rayyan.ai) [51] was employed for duplicate removal and blinded title/abstract screening.

### 3.4. PRISMA Flow and Article Selection

**Figure 1:**
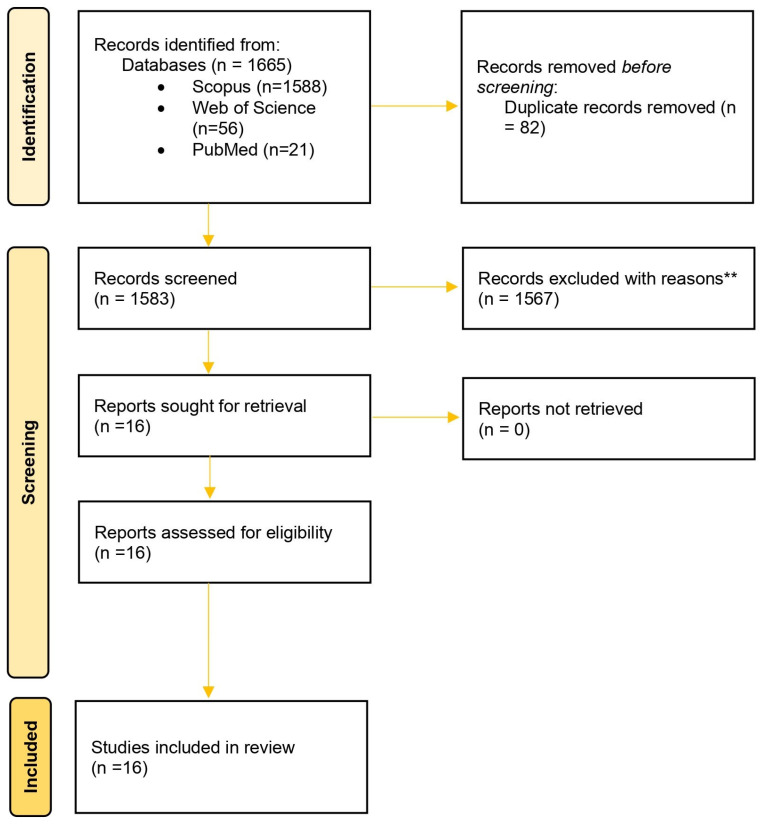
PRISMA (Preferred Reporting Items for Systematic Reviews and Meta-Analyses) 2020 flow diagram highlighting the identification, screening, and inclusion of studies on the fermentation of *Acheta domesticus* powder in food applications. Records were retrieved from Scopus, Web of Science, and PubMed using a predefined search query. The flowchart exhibits the systematic inclusion/exclusion phases to ensure transparency in the selection process in accordance with PRISMA guidelines. The “**” symbol in the box indicates that the records were excluded due to incomplete information on *A. domesticus* fermentation, lack of full-text availability, or not meeting methodological criteria.

To ensure the clarity of the report, a PRISMA flow was followed including three steps: 1—identification; 2—screening; and 3—inclusion. The initial search retrieved 1665 articles across all the databases. A cross-referencing approach was employed to identify relevant studies from the bibliographies of retrieved papers. After removing 82 duplicates, 1583 articles were used for screening. One thousand five hundred sixty-seven articles were excluded at title/abstract level, and 16 full-text articles were evaluated for eligibility. All 16 articles met entire the inclusion criteria and were directly related to the fermentation of *A. domesticus* in food systems (Figure 1).

### 3.5. Data Extraction and Synthesis

The information collected from the literature review covered several key aspects: (i) publication details: authors, year of publication, and journal; (ii) type of food product investigated (e.g., bread, dairy, beverages, or other matrices); (iii) fermentation microorganisms employed (e.g., LAB or yeasts); (iv) processing form of *A. domesticus* powder used (e.g., raw, roasted, defatted, enzymatically treated); (v) effects of fermentation on nutritional composition and functional properties; (vi) sensory and technological properties of the resulting products; and (vii) consumer acceptance of fermented *A. domesticus* powder in foods.

In total, according to the scope of the review, 16 papers were selected (Table 1 and Table 2), summarized through a narrative synthesis, and discussed. To facilitate interpretation, the extracted information was organized by food matrix type (e.g., bakery, dairy, beverages) and by fermentation microorganism category (e.g., LAB, yeasts) to highlight trends, applications, and research gaps in this emerging field.

## 4. Processing and Fermentation Strategies for *A. domesticus*

A comprehensive recent literature review by Yan et al. [65] examined the transformation of edible insects into various commercial formats for human consumption by exploiting the currently available processing techniques. The most common forms include whole dried insects, insect powder, and protein extracts or isolates. Whole dried crickets retain the complete nutritional matrix, including fiber, fats, and chitin, but may present challenges in terms of consumer acceptance due to visual and textural attributes. In contrast, insect powder is more versatile and can be incorporated into a wide range of food products, from baked goods to protein bars, enhancing palatability and masking the insect origin beyond boasting an extended shelf life. Finally, protein extracts, obtained through fractionation or enzymatic hydrolysis, allow for the development of high-protein functional ingredients, suitable for food fortification and specialized dietary applications. Recently, edible insect pastes have also been manufactured and analyzed. These semi-solid formulations preserve much of the insect’s original moisture and structure, allowing for greater retention of certain heat-sensitive nutrients and bioactive compounds.

According to what was highlighted by Yan et al. [65], each formulation presents unique advantages and limitations in terms of processing feasibility, shelf life, nutritional value, and consumer perception, making the selection of an appropriate form critical for the successful integration of *A. domesticus* into mainstream diets, each offering distinct nutritional, functional, and sensory profiles.

To date, several of these insect-based formats—particularly powders and pastes—have been subjected to fermentation processes. Fermentation, traditionally recognized for its pivotal role in food preservation, also represents a versatile biotechnological approach with considerable potential to enhance the nutritional profile, improve the bioavailability of key compounds, and confer functional attributes to insect-derived food matrices. Moreover, this approach can contribute to the modulation of sensory properties and the reduction in antinutritional factors, thereby increasing consumer acceptability and product quality. These aspects are discussed in detail in the following chapters.

## 5. Fermentation of *A. domesticus* as a Promising Biotechnological Approach for Enhancing the Functionality of Cricket-Based Ingredients (Powder or Paste)

The incorporation of *A. domesticus* into food systems is primarily driven by its high protein content and the presence of bioactive compounds. However, concerns related to allergenicity, the formation of process contaminants (e.g., acrylamide), and limited consumer acceptance, especially in Western cultures, present significant challenges [4]. In this context, microbial fermentation has emerged as a powerful biotechnological approach to improve the nutritional value, safety, and sensory characteristics of insect-derived ingredients [66].

To date, several studies have emphasized the role of LAB and yeast in modulating the biochemical properties of cricket-based food ingredients, e.g., powder or paste, as detailed below (Table 1).

Bartkiene et al. [4] evaluated the effect of fermenting cricket powder with *L. plantarum* No. 122 (48 h at 30 °C) on its physicochemical properties and its application in wheat bread. The objective was to assess whether fermentation could enhance bread quality and safety while reducing potential risks associated with insect-based ingredients. Fermentation of cricket powder led to significant biochemical and technological changes. The process resulted in acidification, a reduction in pH, and a substantial increase in viable LAB, confirming the effectiveness of the starter culture. Fermented samples displayed modified color parameters and a decrease in total biogenic amine content, particularly for putrescine and cadaverine, although tyramine levels increased with extended fermentation. The treatment also influenced the fatty acid profile and generated organic acids such as oleic (26.28% increase after 48 h of fermentation), lactic, acetic, and succinic acids, contributing to improved microbial safety. When incorporated into bread, fermented cricket powder increased specific volume and porosity compared to non-fermented controls, and crumb and crust colors were modified in line with Maillard reaction intensities. Importantly, breads containing 10% fermented cricket powder showed lower acrylamide content than those with untreated powder, highlighting a positive safety effect, whereas higher inclusion levels did not yield the same benefit. Sensory analysis indicated that the addition of fermented cricket powder induced favorable consumer responses, with higher associations to positive emotions such as “happy.” Overall, fermentation with *L. plantarum* No. 122 improved digestibility, microbial quality, and some safety attributes, supporting its use as a pre-treatment strategy for the inclusion of cricket powder in breadmaking.

Vasilica et al. [2] similarly showed that fermentation of *A. domesticus* powder by *L. plantarum* (48 h at 37 °C) influenced organic acid profiles, fatty acid content, amino acid release, aroma compounds, and mineral bioavailability. Citric acid was consumed as an alternative source of energy by LAB strains under low-carbohydrate conditions, which resulted in its reduction and a simultaneous increase in succinic acid. Fermentation also increased fatty acids, particularly polyunsaturated fatty acids (PUFAs), according to LAB-mediated lipid transformations such as isomerization, hydration, and lipolysis. These lipid transformations also participated in the production of aldehydes, ketones, and alcohols through lipid oxidation and amino acid catabolism. Moreover, fermentation led to aroma development by producing compounds like 3-methyl-1-butanol and 2-methyl-5-propan-2-ylcyclohex-2-en-1-one. Amino acid content increased due to proteolytic activity and protein degradation during 24 h of *L. plantarum* fermentation, particularly Ala (1.76-fold), Gly (3.67-fold), Leu (1.99-fold), and Met (2.89-fold). Furthermore, mineral bioavailability and decreased pH activated phytase enzymes and reduced anti-nutritional factors like phytates and tannins.

Yeast fermentation has also emerged as a promising biotechnological approach for enhancing the functionality of cricket-based ingredients.

Rossi et al. [52] explored the use of *Yarrowia lipolytica* RO25 to hydrolyze cricket powder and evaluate its suitability as an ingredient for sourdough production. The resulting hydrolysate was tested in sourdough systems together with selected LAB to assess microbiological, biochemical, and sensory parameters. Fermentation of cricket powder with *Y. lipolytica* (72 h at 25 °C) produced a hydrolysate characterized by intense proteolytic and lipolytic activities, which significantly modified the protein and lipid composition of the matrix. Compared to non-hydrolyzed cricket powder, the hydrolyzed version promoted distinctive protein patterns, particularly in the glutelin fraction, and enhanced the release of small peptides. Moreover, the fermentation process markedly increased the amount of free fatty acids, including arachidonic and linolenic acids, which are associated with functional and health-promoting properties. Volatile profiles revealed a higher abundance of aldehydes and ketones, indicating that the hydrolysate contributed precursors for desirable aroma compounds. Importantly, the microbial system produced sourdoughs with specific sensory fingerprints and improved functional potential compared to wheat-only controls. Overall, the findings demonstrate that fermentation with *Y. lipolytica* RO25 transforms cricket powder into a more suitable ingredient for sourdough, with enhanced nutritional quality and unique aromatic properties.

Expanding on this approach, Patrignani et al. [53] evaluated the use of the strains *Y. lipolytica* PO11 and *Debaryomyces hansenii* DB for cricket powder hydrolysis (72 h at 25 °C). The microbiological quality of raw cricket powder was high, and low counts of spoilage organisms were observed. *Y. lipolytica* PO11 showed a growth of over 8 log CFU/g within 24 h. *D. hansenii* DB reduced pH to 5.5 according to acetic acid production. Fermentation also enhanced protein and free amino acid content by increasing glutamic acid, serine, and threonine in crickets fermented by *D. hansenii* DB, and leucine, isoleucine, methionine, and cysteine in crickets fermented by *Y. lipolytica* RO25. Moreover, a general increase in protein content after fermentation was observed, with a peak at 48 h for *D. hansenii* SPL612 (26.35% increase). Of note, bioactive compounds like γ-aminobutyric acid (GABA) and β-aminobutyric acid (BABA) were detected in samples fermented by *Y. lipolytica* PO11 and *Y. lipolytica* RO25, which led to enhancement of hydrolysates’ functional properties such as improved digestibility and reduced chitin content. Fatty acids developed by *Y. lipolytica* strains included unsaturated fatty acids such as C18:3 and C20:4, and free fatty acids like C18:2 and C18:1. Furthermore, volatile compound identification using gas chromatography–mass spectrometry (GC-MS) reported over 80 aroma molecules such as alcohols, esters, acids, ketones, pyrazines, sulfur compounds, furanones, and thiophenes [53]. Overall, fermentation transformed cricket powder into hydrolysates, demonstrating the potential of these yeasts to produce innovative and sustainable food ingredients.

In another study, Rossi et al. [54] reported the production of a cricket-based bread using *Y. lipolytica* R025 hydrolyzed cricket sourdough. *Y. lipolytica* RO25 hydrolysis resulted in the reduction in chitin, improving rheology, and producing breads with hardness like wheat control bread. Furthermore, gumminess, cohesiveness, and chewiness were not affected across the samples. The *Y. lipolytica* RO25-hydrolyzed cricket bread demonstrated a high concentration of polyunsaturated free fatty acids, protein content (specially albumins/globulins, prolamins, and glutelins) and a diverse volatile compound profile by identification of more than 120 molecules. Additionally, biogenic amine levels were supposed to be lower than the bread made with non-hydrolyzed cricket powder which contained highest tyramine levels. Sensory evaluation also revealed a texture and flavor enhancement of *Y. lipolytica* RO25-hydrolyzed cricket bread.

## 6. Fermentation-Driven Modifications in Fermented Foods Enriched with *A. domesticus*

Over the past two decades, the exploitation of *A. domesticus* for the production of various food formulations has been the subject of extensive research. Numerous studies summarized in recent literature reviews have examined the effects of cricket incorporation on the nutritional, functional, and sensory properties of a wide range of food products. These reviews clearly highlighted a growing scientific consensus on the potential of cricket to improve food quality while aligning with environmental and sustainability objectives. However, they have largely overlooked the role of fermentation in modulating the characteristics of insect-enriched foods. To address this gap, the present review provides an in-depth analysis of how fermentation influences nutritional value, safety, and sensory attributes of fermented foods containing *A. domesticus*, thereby offering new perspectives on the development of sustainable and palatable fermented insect-based products (Table 2).

One of the most extensively studied applications of fermentation for the production of cricket-enriched foods is undoubtedly breadmaking.

In 2023, Bartkiene and colleagues [55] used cricket powder fermented by *L. plantarum* in wheat bread at 10–30% inclusion levels. Fermentation of cricket powder by *L. plantarum* reduced pH values and supported high bacterial counts, confirming its suitability as a fermentable substrate. The process modified color attributes and led to a decrease in total biogenic amine content (13.1%), with a marked reduction in compounds such as cadaverine and putrescine. At the same time, fermentation promoted the appearance of volatile metabolites including acids, alcohols, and aldehydes, contributing to a more complex aroma profile. When incorporated into bread, fermented cricket powder improved loaf volume and crumb porosity compared with non-fermented samples. Moreover, the use of fermented powder lowered acrylamide levels in some formulations, thus enhancing product safety. Sensory evaluations indicated that overall acceptability remained high, while breads with fermented cricket powder generated more positive emotional responses. These findings demonstrate that fermentation can be an effective pre-treatment to improve the technological and nutritional potential of insect-based bakery products [55].

A study by Osimani et al. [3] aimed to evaluate the technological, microbiological, and nutritional impact of adding *A. domesticus* powder (10–30%) to bread formulations. The objective was to clarify how sourdough fermentation modifies the composite matrix and affects the overall quality of the final product. In the study by Osimani et al. [3] fermentation of doughs enriched with cricket powder led to relevant changes in acidity, as shown by higher titratable acidity compared with controls, a trend linked to the ash content of the insect ingredient and its buffering capacity. Microbiological analysis revealed that LAB remained metabolically active in these matrices, contributing to acid production and helping to stabilize the dough ecosystem. The use of sourdough also limited the proliferation of undesirable microorganisms, although the persistence of spore-forming bacteria highlighted potential safety concerns requiring targeted pre-treatments. From a technological perspective, the incorporation of cricket powder reduced gluten-network strength and increased dough development time, particularly at higher substitution levels. This resulted in breads with lower specific volume and firmer crumb texture, although products with 10% inclusion remained suitable for breadmaking. Fermentation further influenced protein breakdown and amino acid profiles, enhancing levels of essential amino acids such as lysine and valine. Volatile compound analysis showed that sourdough activity enriched the aroma profile through the release of acids, alcohols, and aldehydes, while cricket-derived fractions contributed unique notes. Sensory evaluation indicated that breads with moderate enrichment were acceptable to consumers, especially when insects were not visible in the product. Overall, sourdough fermentation improved nutritional features and modulated the technological behavior of cricket-based doughs, supporting the potential of this novel ingredient in functional bakery applications.

Fermented cricket powder has also been evaluated in gluten-free baking. A study by Kowalczewski et al. [56] incorporated 2–10% cricket powder into gluten-free bread, leading to substantial increases in protein, minerals (Cu, P, Zn), polyphenols, and antioxidant activity. Interestingly, gut health markers, such as *β*-glucuronidase activity, were positively modulated, and no negative impact was observed on intestinal microflora.

Nissen et al. [57] investigated the effects of sourdough fermentation on gluten-free bread enriched with *A. domesticus* powder. The objective was to evaluate changes in nutritional quality, antioxidant capacity, and aromatic profile. Fermentation driven by LAB, mainly *L. plantarum* and *Lactobacillus sanfranciscensis*, enhanced microbial activity in cricket-enriched doughs and stimulated the production of organic acids and alcohols, shaping a distinctive volatilome. Compared with controls, breads containing cricket powder displayed a more complex aroma and significantly higher antioxidant properties, particularly after LAB fermentation. These results indicate that sourdough processing improves both nutritional value and sensory traits of cricket-based gluten-free products.

Galli et al. [58] aimed to characterize the microbial community of *A. domesticus* powder and to evaluate the effect of LAB fermentation on its application in breadmaking. The objective was to identify suitable starter strains and assess their influence on dough and bread quality. In this study, spontaneous fermentation of cricket powder allowed the isolation of several LAB species, including *L. plantarum*, *L. curvatus*, *L. sakei*, *Lactococcus garvieae*, *W. confusa*, and *Enterococcus durans*. Among them, *L. plantarum* CR L1 and *L. curvatus* CR L13 showed superior performance in terms of acidification, peptidase activity, and robustness during backslopping, making them promising starters for sourdough. When used in cricket–wheat sourdough, these strains ensured high microbial stability, effective acidification, and consistent cell growth. Although the inclusion of cricket powder reduced dough volume compared to wheat-only controls, breads enriched with 20% cricket powder exhibited a substantial increase in protein (over 80%) and lipid content. Overall, fermentation improved the nutritional profile of the breads and demonstrated the potential of cricket powder combined with lactic fermentation as a novel ingredient for functional bakery products.

A study carried out by Cappelli et al. [59] aimed to evaluate the technological performance of wheat dough enriched with *A. domesticus* powder (5–15%). The objective was to investigate how the partial replacement of wheat flour affects dough rheology and bread characteristics. The addition of cricket powder influenced water absorption and dough development, with higher substitution levels leading to increased protein content and changes in stability. At 15% inclusion of cricket powder, dough stability improved and softening decreased, indicating a stronger structure. However, higher protein content also reduced extensibility, limiting gas retention and bread volume. As a result, breads made with cricket powder showed higher crumb density and lower specific volume compared with the wheat control. Despite these technological drawbacks, the inclusion of cricket powder substantially enhanced protein content, confirming its potential for nutritional enrichment. Overall, moderate substitution levels (5–10%) offered a balance between acceptable bread quality and improved nutritional value.

Belleggia et al. [60] aimed to investigate the role of sourdough fermentation in flatbreads enriched with cricket (*A. domesticus*) powder (20%), examining its influence on microbiological stability, physicochemical traits, and sensory quality. The purpose was to clarify how fermentation reshapes the food matrix when insect-based ingredients are introduced. When cricket powder was incorporated into dough and subjected to sourdough fermentation, relevant modifications were observed. Fermented doughs showed an active community of LAB, including *Lactobacillus* species and *Pediococcus*, which drove acidification and raised titratable acidity compared to controls. The buffering effect of the insect-derived proteins and minerals supported consistent fermentation and moderated pH changes. In the baked products, sourdough contributed to safer microbial profiles and maintained water activity at levels unfavorable for spoilage. The structural role of chitin, interacting with fermentation by-products, led to softer textures and reduced hardness of the flatbreads. In parallel, the metabolic activity of sourdough microorganisms enhanced the release of volatile compounds, broadening the aroma spectrum with alcohols, acids, aldehydes, and ketones. Some of these volatiles appeared specifically linked to the cricket fraction, underlining its contribution to flavor complexity. Overall, sourdough fermentation not only stabilized the insect-based matrix but also improved sensory properties, demonstrating the suitability of cricket powder as a functional ingredient in innovative bakery goods. However, there is still a need for consumer acceptance studies and nutritional evaluation to support their market acceptability.

Beyond bakery applications, cricket has also been explored for manufacturing fermented dairy products, beverages, and seasoning sauces.

Concerning dairy products, Karwacka et al. [61] examined the feasibility of incorporating *A. domesticus* powder (1.5–5%) into yoghurt formulations. The objective was to analyze its impact on fermentation, physicochemical properties, and sensory quality. Fermentation carried out with *Lactobacillus delbrueckii* subsp. *bulgaricus* and *Streptococcus thermophilus* was not hindered by the addition of cricket powder. The enriched yoghurts showed higher protein (7.83%) and fat (5.18%) contents, faster acidification in some variants, and significant modifications in texture and color. Overall, lactic fermentation ensured microbial stability while cricket powder enhanced nutritional value, although high inclusion levels reduced consumer acceptance.

In 2023, Dridi et al. [62] evaluated protein hydrolysates from fermented cricket powder (using *Lactobacillus acidophilus* CL1285, *L. casei* LBC80R, and *Lactobacillus rhamnosus* CLR2). In vivo digestibility trials in rats over 14 days (n = 7 per group) showed improved protein efficiency ratio (PER), food intake, and growth rates, especially for hydrolyzed forms with a true digestibility of 94.0 ± 0.8%, compared with 96.6 ± 0.3% for casein control and 85.8 ± 1.5% for the whole cricket powder diet (*p* ≤ 0.05). These results confirmed the suitability of cricket protein for use in high-performance functional drinks.

Regarding sauces, Kittibunchakul et al. [63] investigated the processing of *A. domesticus* into a fermented cricket paste by mimicking the traditional preparation of Thai shrimp paste, Kapi. The objective was to evaluate the physicochemical, microbiological, and nutritional changes induced by fermentation. Fermentation, initiated with Kapi as starter culture, led to the proliferation of LAB as predominant microorganisms, accompanied by halophilic bacteria adapted to high salt levels. These microbial activities reduced pH, supported proteolysis, and generated peptides and amino acids contributing to flavor and texture development. Salting and drying steps lowered water activity to ~0.7, enhancing microbial safety and extending shelf-life without refrigeration. Protein digestibility significantly improved in the fermented cricket paste compared to raw crickets (93.8% vs. 81.9%). The amino acid profile showed an increased proportion of essential amino acids (11.18%) and was mainly composed of glutamic acid, aspartic acid, and alanine. Despite reduced overall macronutrient content due to processing losses, fermentation enhanced protein quality and digestibility, suggesting the fermented cricket paste as a promising protein-rich seasoning and alternative to fermented shrimp paste.

More recently Dhakal et al. [64] have also reported the production of a fermented seasoning sauce from *A. domesticus* using salt-tolerant proteolytic bacteria (*Staphylococcus piscifermentans* TISTR 824 and *Halobacillus* sp. TISTR 1860) in a combined process of enzymatic digestion and fermentation. The resulting fermented sauce demonstrated physiochemical properties (degree of hydrolysis, pH, absorbance at 420 nm, and water activity) like traditional Thai fish sauces but with a unique volatile flavor profile. It also exhibited an increase in antioxidant potential compared to the raw cricket meal. Moreover, bioactivity was enhanced according to the release of bioactive peptides during the production process. The fermented sauce showed higher in vitro anti-diabetic effects than the commercial Thai fish sauce and was classified as “light in sodium”.

## 7. Consumer Acceptance Challenges for Fermented Cricket-Enriched Food Ingredients and Food Products

As already stated, despite the well-documented nutritional, functional, and environmental advantages associated with edible insects—particularly *A. domesticus*—their integration into Western diets continues to face substantial barriers. These challenges encompass consumer perception, food safety concerns, suboptimal sensory characteristics, and the limited scalability of production and processing systems. Among these, consumer acceptance remains a pivotal bottleneck. In Western societies, where entomophagy is culturally alien and often elicits aversion or perceived risk, psychological resistance is influenced by multiple socio-demographic variables, including age, gender, education, and food neophobia. Younger, more food-adventurous consumers typically demonstrate greater openness to insect-based foods, whereas older or neophobic individuals are more reluctant. These insights underline the importance of developing tailored communication strategies and designing products that resonate with the expectations and preferences of specific consumer groups.

From a technological standpoint, processing methods, particularly fermentation, have emerged as promising tools to bridge the gap between product development and consumer acceptance. Fermentation not only serves to modulate microbial safety but also significantly enhances sensory properties such as flavor, texture, and aroma. As emphasized by Okaiyeto et al. [12], these improvements can attenuate negative sensory perceptions commonly associated with insect-based ingredients.

Furthermore, consumer acceptability can be increased by employing multi-pronged approaches: (i) incorporating insects in non-visible or familiar food formats (e.g., bread, biscuits, yogurt); (ii) using flavor-masking or -enhancing compounds; (iii) educating consumers on sustainability and nutritional benefits; and (iv) leveraging fermentation not only as a processing method but also as a vehicle for introducing beneficial microbial cultures, potentially derived from the insects themselves.

Recent research has also highlighted the complex interplay between sensory properties and emotional responses. Bartkiene et al. [55] demonstrated that white bread enriched with up to 30% cricket powder exhibited increased sourness, bitterness, and darker coloration, yet overall consumer acceptability was maintained. Interestingly, higher inclusion levels were associated with stronger emotional responses, both positive (e.g., “happy”) and negative (e.g., “sad”), likely driven by specific volatile compound profiles. Notably, the addition of 5% cricket powder resulted in no significant compromise to sensory quality or consumer approval, suggesting that low-to-moderate incorporation levels can be effective for market entry formulations.

In this context, fermentation stands out not only as a functional biotechnological intervention but also as a consumer-centric innovation strategy, capable of aligning technological feasibility with perceptual acceptability in the development of cricket-enriched food products.

## 8. Conclusions and Future Perspectives

**Figure 2:**
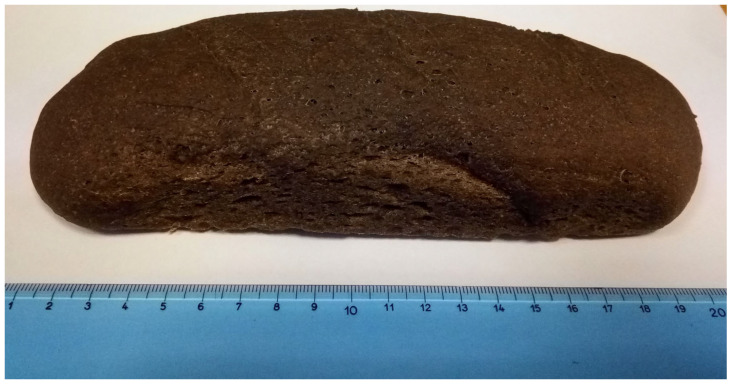
Cricket-enriched bread, exhibiting the incorporation of fermented cricket powder in a baked product.

*A. domesticus* represents a promising frontier in sustainable nutrition. From the analysis of the available scientific literature, it emerges that fermentation and related biotechnological processes represent powerful tools to enhance nutritional, functional and sensory profiles of cricket-based ingredients and foods. Of note, bakery products (Figure 2), especially bread and biscuits, have been the most studied matrices, followed by fermented beverages and dairy alternatives.

As emerged by the analysis of the available literature, *A. domesticus* powder or protein hydrolysates were suggested to lead to nutritional enhancements to food products such as bread, biscuits, cookies, and beverages. Most commonly, the incorporation of cricket powder or protein hydrolysates resulted in an increase in protein content and a more complete essential amino acid profile. Notably, fermentation using *Y. lipolytica* is supposed to increase the content of polyunsaturated fatty acids (PUFAs) such as arachidonic and linoleic acids. Moreover, fatty acids aroma precursors such as C18:1 (oleic acid), C18:2 (linoleic acid), and C20:4 (arachidonic acid) were present in higher levels in the produced hydrolysates. The employment of *Y. lipolytica* and *D. hansenii* also demonstrated a reduction in chitin, production of antimicrobial substances, and increased matrix digestibility.

Interestingly, the use of *L. plantarum* and *L. casei* led to an increase in oleic, palmitic, and linoleic acids in fermented cricket powder. Moreover, fermentation with *L. plantarum* resulted in a rise in saturated and monounsaturated fatty acids, accompanied by a decrease in polyunsaturated fatty acids. These diverse changes in fatty acid composition were in accordance with the type of microbial strain used and fermentation conditions. Furthermore, LAB fermentation not only improved the nutritional profile, but also led to improved safety and detoxification of food products by reductions in acrylamide, biogenic amines, and lipid oxidation. Fermentation with *L. plantarum* also increased the content of organic acids such as lactic, acetic, and oxalic acids, improved amino acid availability, and modified fatty acid composition. Furthermore, microbial metabolism also played a role in reducing anti-nutritional compounds like phytates and tannins, which led to improving nutritional features of the cricket-based ingredient.

Of note, an increase in mineral bioavailability was also observed in foods fermented by yeasts or LAB. Although a few studies investigated the sensory effects of fermented cricket-enriched foods, some of them have pointed out darker coloration and softer textures in fermented or hydrolyzed cricket products. A reduction in lightness and yellowness in fermented cricket powder was also observed. Interestingly, in yoghurt, the addition of cricket powder decreased texture firmness and brightness, potentially affecting consumer perception. Ultimately, it is important to consider that baked products containing no more than 10% of cricket powder proved to have more acceptable sensory properties in cricket-based products. Hence, the need for a proper dosage of cricket-based ingredients in the cricket-enriched food recipes is recommended.

Notably, a richness in volatile compounds was observed in fermented cricket-enriched foods that showed the presence of 60 to more than 80 different molecules. Among the major compounds, alcohols, aldehydes, esters, sulfur compounds, pyrazines, and other compounds were included. Interestingly, fermentation with *L. plantarum* led to the formation of acetic acid, hexanal, and decane, as well as the production of pleasant aromas such as benzylaldehyde and 3-methyl-1-butanol. Those volatiles, deriving from protein hydrolysis, where responsible for the unique flavors characterizing the obtained fermented food. Finally, fermentation has helped improve textural stability, lightness, and water activity control particularly in bread and seasoning sauces. Moreover, cricket-based fermented pastes exhibited antimicrobial activity and lower lipid oxidation, thus suggesting that fermentation had a positive role in enhancing both preservation and safety of the end-product.

As a final remark, it is expected that, through interdisciplinary collaboration, technological innovation, and strategic consumer engagement, *A. domesticus* can transition from niche novelty to mainstream protein source, meaningfully contributing to global food security and environmental sustainability.

Although the limited number of existing studies represents a constraint, this literature review provides a valuable foundation for future investigations and practical applications. Overall, this work contributes to advancing understanding in an emerging and promising field.

## Figures and Tables

**Table 1:** Case studies on the role of lactic acid bacteria (LAB) and yeasts on biochemical enhancement of cricket-based food ingredients.

Type of Fermented Ingredient	Microorganism Used	Obtained Enhancement	Reference
*Acheta domesticus* powder	*Lactiplantibacillus. plantarum* and *Lacticaseibacillus casei* (48 h at 30 °C)	**Nutritional**: - Enhancing fatty acid profile by increasing linoleic, oleic, palmitic, and linolenic acids, with the highest value of 26.28% increase after 48 h for oleic acid. - Decrease in biogenic amines by a decrease in cadaverine and putrescine. **Sensory**: - Increase in volatile compounds such as acetoin and 3-methylbutanoic acid and decrease in hexanal. - Decrease in lightness and yellowness. **Functional**: n/a	Bartkiene et al. [4]
*A. domesticus* powder	*L. plantarum* (48 h at 37 °C)	**Nutritional**: - Organic acid profile improvement by reduction in citric acid and an increase in succinic acid - Increase in amino acid content particularly Ala (1.76-fold), Gly (3.67-fold), Leu (1.99-fold), and Met (2.89-fold). - Increase in fatty acids particularly PUFAs (polyunsaturated fatty acids) - Reduction in anti-nutritional factors like tannins and phytates. **Sensory**: - Production of aroma compounds such as aldehydes, ketones, and alcohols. **Functional**: n/a	Vasilica et al. [2]
Cricket powder-based hydrolysate	*Yarrowia lipolytica* (72 h at 25 °C)	**Nutritional**: - Rise in fatty acid content especially arachidonic and linoleic acids. - High release of volatile precursors such as C18:1, C18:2 and C20:4 **Sensory**: - Identification of around 60 volatile compounds mostly aldehydes and ketones. **Functional**: n/a	Rossi et al. [52]
Cricket powder-based hydrolysate	*Y. lipolytica* and *Debaryomyces hansenii* (72 h at 25 °C)	**Nutritional:** - Significant increase in protein content, with the highest value of 26.35% after 48 h for *D. hansenii* SPL612. - Reduction in chitin content. - Increase in antimicrobial substances and health-promoting molecules. - Detection of bioactive compounds like γ-aminobutyricacid (GABA) and β-aminobutyricacid (BABA). - Increased fatty acid profile, especially unsaturated fatty acids. - High matrix digestibility due to the release of amino acids. **Sensory**: - Identification of over 80 aroma compounds such as alcohols, ketones, and pyrazines. **Functional**: n/a	Patrignani et al. [53]
Cricket powder-based hydrolysate	*Y. lipolytica*	**Nutritional:** - Reduction in chitin content. - High protein content. - High concentration of polyunsaturated free fatty acids. - Reduction in biogenic amine levels compared to the non-hydrolyzed breads. **Sensory**: - Possessing a diverse volatile compound profile by identification of more than 120 molecules. - Enhanced texture and flavor. **Functional**: - Improvement of rheology.	Rossi et al. [54]

n/a: not available.

**Table 2:** Case studies on cricket-based fermented food products by using lactic acid bacteria (LAB) and yeasts species.

Product Type (% Inclusion)	Form of Cricket Used	Microorganism Used	Obtained Enhancement	Reference
Cricket-enriched bread (10, 20, 30%)	*Acheta domesticus* powder	*Lactiplantibacillus plantarum*	**Nutritional**: - Fatty acids profile enhancement by increasing in saturated and monosaturated fatty acids, decreasing in polyunsaturated fatty acids. - Reduction in biogenic amines (13.1%). - Disappearance of cadaverine and putrescine after 48 h. **Sensory**: - Increase in volatile compounds like acetoin, 2,3-butanediol, and pyrazines. - Decrease in lightness, redness, and yellowness. **Functional**: n/a	Bartkiene et al. [55]
Cricket-powder enriched sourdough bread (10, 30%)	*A. domesticus* powder	*Lactobacillus sanfranciscensis* PB276, *L. sanfranciscensis* PB223, *Lactobacillus plantarum* PB11, *L. plantarum* PB24, *Lactobacillus fermentum* PB162	**Nutritional**: - Increase in protein content. - High amount of essential amino acids. - Improved fatty acid profile. **Sensory**: n/a **Functional**: n/a	Osimani et al. [3]
Cricket-enriched gluten-free bread (2, 6, 10%)	*A. domesticus* powder	n/a	**Nutritional**: -Increase in protein content. - Higher mineral content. - Increase in antioxidant activities and polyphenols. - Decreasing markers activities such as β-glucuronidase. - No inhibitory effect on the growth of microflora. **Sensory**: n/a **Functional**: n/a	Kowalczewski et al. [56]
Cricket-fortified gluten-free sourdough bread (n/a %)	*A. domesticus* powder	*L. plantarum* 98a, *L. sanfranciscensis* Bb12 and *Saccharomyces cerevisiae* LBS	**Nutritional**: -Increased antioxidant activity by protein hydrolysates. - Decrease in lipid oxidation by antioxidants. - Unique volatile compounds profile. **Sensory**: n/a **Functional**: n/a	Nissen et al. [57]
Cricket-enriched bread (20%)	Cricket powder	*L. plantarum* CR L1, *Latilactobacillus curvatus* CR L13, *Lactococcus* spp., *Enterococcus* spp., and *Weissella* spp.	**Nutritional**: - Higher protein and fat content in bread. - Identification of *L. plantarum* and *L. cuvatus* as suitable microbial starters. **Sensory**: n/a **Functional**: n/a	Galli et al. [58]
Cricket-enriched bread (5, 10, 15%)	*A. domesticus* powder	n/a	**Nutritional**: n/a **Sensory**: n/a **Functional**: - Increase in stability. - Reduction in softening.	Cappelli et al. [59]
Cricket-enriched flatbread (20%)	*A. domesticus* powder	Lactic acid bacteria and yeasts naturally contained in the sourdough used as leavening agent or baker’s yeast	**Nutritional:** - Identification of numerous aromatic compounds (more than 540) such as alcohols, aldehydes, esters, ketones, acids, pyrazines, furans, and sulfur compounds. **Sensory:** - Softer texture of flatbreads due to the fiber content. - Darker color of breads which could be advantageous in consumer perception. **Functional:** - Low water-activity of flatbreads, preventing growth of pathogenic microorganisms.	Belleggia et al. [60]
Cricket-enriched yoghurt (1.5, 3, 5%)	*A. domesticus* powder	*Lactobacillus delbrueckii* subsp. *bulgaricus* and *Streptococcus thermophilus*	**Nutritional**: - Increase in protein (7.83%) and fat (5.18%) content of the yoghurt. **Sensory**: - Negatively affecting texture, appearance, and losing hardness and consistency. **Functional**: n/a	Karwacka et al. [61]
Beverage (n/a %)	Cricket powder	*Lactobacillus acidophilus* CL1285, *Lactobacillus casei* LBC80R, and *Lactobacillus rhamnosus CLR2*	**Nutritional**: - Improved protein efficiency. - Complete amino acid profile. - Improvement of the growth parameters, food intake and protein efficiency ratio. **Sensory**: - Higher digestibility (94%) according to the presence of cricket protein hydrolysates. **Functional**: n/a	Dridi et al. [62]
Cricket paste (n/a %)	*A. domesticus* powder	Microorganisms naturally contained in Kapi used as starter	**Nutritional**: - Increase in total essential amino acids (11.18%) compared to the whole cricket powder. - Flavor development due to protein hydrolysis. **Sensory**: - Soft and pasty texture. - Unique flavor and texture properties due to the protein hydrolysis. - Increase in lightness after salting. **Functional**: - Decrease in moisture content. - Lower water activity.	Kittibunchakul et al. [63]
Seasoning sauce (n/a %)	*A. domesticus*	*Staphylococcus piscifermentans* TISTR 824 and *Halobacillus* sp. TISTR 1860	**Nutritional**: - Physiochemical properties such as degree of hydrolysis, pH, absorbance at 420 nm and water activity. - Unique flavor profile - Increase in antioxidant potential. - Enhanced bioactivity due to the release of bioactive peptides. - High anti-diabetic effects compared to Thai fish sauce. **Sensory**: n/a **Functional**: n/a	Dhakal et al. [64]

n/a: not available.

## Data Availability

The original contributions presented in this study are included in the article. Further inquiries can be directed to the corresponding author.

## Abbreviations

The following abbreviations are used in this manuscript:

BABA	β-aminobutyric acid
CBE	Circular Bioeconomy
CFU	Colony-Forming Units
DPPH	2,2-diphenyl-1-picrylhydrazyl
EFSA	European Food Safety Authority
EU	European Union
GABA	γ-aminobutyric acid
GC-MS	Gas Chromatography-Mass Spectrometry
HDM	House Dust Mite
LAB	Lactic Acid Bacteria
NGS	Next-Generation Sequencing
PER	Protein Efficiency Ratio
PUFA	Polyunsaturated fatty acids
ROS	Reactive Oxygen Species
TNF-α	Tumor Necrosis Factor
WHC	Water-Holding Capacity

## References

[1] Pilco-Romero G., Chisaguano-Tonato A.M., Herrera-Fontana M.E., Chimbo-Gándara L.F., Sharifi-Rad M., Giampieri F., Battino M., Vernaza M.G., Álvarez-Suárez J.M. (2023). House Cricket (*Acheta domesticus*): A Review Based on Its Nutritional Composition, Quality, and Potential Uses in the Food Industry. Trends Food Sci. Technol..

[2] Vasilica B.B., Chiș M.S., Alexa E., Pop C., Păucean A., Man S., Igual M., Haydee K.M., Dalma K.E., Stănilă S. (2022). The Impact of Insect Flour on Sourdough Fermentation-Fatty Acids, Amino-Acids, Minerals and Volatile Profile. Insects.

[3] Osimani A., Milanović V., Cardinali F., Roncolini A., Garofalo C., Clementi F., Pasquini M., Mozzon M., Foligni R., Raffaelli N. (2018). Bread Enriched with Cricket Powder (*Acheta domesticus*): A Technological, Microbiological and Nutritional Evaluation. Innov. Food Sci. Emerg. Technol..

[4] Bartkiene E., Zokaityte E., Kentra E., Starkute V., Klupsaite D., Mockus E., Zokaityte G., Cernauskas D., Rocha J.M., Guiné R.P.F. (2023). Characterisation of Lacto-Fermented Cricket (*Acheta domesticus*) Flour and Its Influence on the Quality Parameters and Acrylamide Formation in Wheat Biscuits. Fermentation.

[5] Vehar A., Potočnik D., Mencin M., Korošec M., Ferjančič B., Jagodic Hudobivnik M., Jamnik P., Ota A., Kouřimská L., Kulma M. (2025). Evaluation of Nutritional Quality and Oxidation Stability of Fermented Edible Insects. Foods.

[6] Borrego-Ruiz A., González-Domenech C.M., Borrego J.J. (2024). The Role of Fermented Vegetables as a Sustainable and Health-Promoting Nutritional Resource. Appl. Sci..

[7] Carpentier J., Abenaim L., Luttenschlager H., Dessauvages K., Liu Y., Samoah P., Francis F., Caparros Megido R. (2024). Microorganism Contribution to Mass-Reared Edible Insects: Opportunities and Challenges. Insects.

[8] Hamam M., D’Amico M., Di Vita G. (2024). Advances in the insect industry within a circular bioeconomy context: A research agenda. Environ. Sci. Eur..

[9] Nguyen T.H., Wang X., Utomo D., Gage E., Xu B. (2025). Circular bioeconomy and sustainable food systems: What are the possible mechanisms?. Clean. Circ. Bioecon..

[10] Min Y.R., Nam J.K., Jang H.W. (2025). Edible insects as sustainable food sources: Extraction techniques, nutritional profiles, and volatile characteristics. Anal. Sci. Technol..

[11] Van Campenhout L. (2021). Fermentation technology applied in the insect value chain: Making a win-win between microbes and insects. J. Insects Food Feed.

[12] Okaiyeto S.A., Yu S.H., Deng L.Z., Wang Q.H., Sutar P.P., Wang H., Zhao J.H., Mujumdar A.S., Ni J.B., Lv W. (2024). How to enhance the acceptability of insects food—A review. Food Front..

[13] Alejandro Ruiz F.E., Ortega Jácome J.F., Tejera E., Alvarez-Suarez J.M. (2025). Edible insects as functional foods: Bioactive compounds, health benefits, safety concerns, allergenicity, and regulatory considerations. Front. Nutr..

[14] Belhadj Slimen I., Yerou H., Ben Larbi M., M’Hamdi N., Najar T. (2023). Insects as an Alternative Protein Source for Poultry Nutrition: A Review. Front. Vet. Sci..

[15] Hassan S.A., Altemimi A.B., Hashmi A.A., Shahzadi S., Mujahid W., Ali A., Bhat Z.F., Naz S., Nawaz A., Abdi G. (2024). Edible Crickets as a Possible Way to Curb Protein-Energy Malnutrition: Nutritional Status, Food Applications, and Safety Concerns. Food Chem. X.

[16] Jankowski W.M., Przychodniak D., Gromek W., Majsiak E., Kurowski M. (2025). Edible Insects as an Alternative Source of Nutrients: Benefits, Risks, and the Future of Entomophagy in Europe—A Narrative Review. Foods.

[17] Kemsawasd V., Inthachat W., Suttisansanee U., Temviriyanukul P. (2022). Road to The Red Carpet of Edible Crickets through Integration into the Human Food Chain with Biofunctions and Sustainability: A Review. Int. J. Mol. Sci..

[18] Ma Z., Mondor M., Goycoolea Valencia F., Hernández-Álvarez A.J. (2023). Current State of Insect Proteins: Extraction Technologies, Bioactive Peptides and Allergenicity of Edible Insect Proteins. Food Funct..

[19] Nachtigall L., Grune T., Weber D. (2025). Proteins and Amino Acids from Edible Insects for the Human Diet—A Narrative Review Considering Environmental Sustainability and Regulatory Challenges. Nutrients.

[20] Van Peer M., Berrens S., Coudron C., Noyens I., Verheye G.R., Van Miert S. (2024). Towards Good Practices for Research on *Acheta domesticus*, the House Cricket. J. Insects Food Feed.

[21] Rossi G., Psarianos M., Ojha S., Schlüter O.K. (2025). Insects as a Novel Feed Ingredient: Processing Technologies, Quality and Safety Considerations. Animal.

[22] Tarahi M., Aghababaei F., McClements D.J., Pignitter M., Hadidi M. (2025). Bioactive Peptides Derived from Insect Proteins: Preparation, Biological Activities, Potential Applications, and Safety Issues. Food Chem..

[23] Ververis E., Boué G., Poulsen M., Pires S.M., Niforou A., Thomsen S.T., Tesson V., Federighi M., Naska A. (2022). A Systematic Review of the Nutrient Composition, Microbiological and Toxicological Profile of *Acheta domesticus* (House Cricket). J. Food Compos. Anal..

[24] Pan J., Xu H., Cheng Y., Mintah B.K., Dabbour M., Yang F., Chen W., Zhang Z., Dai C., He R. (2022). Recent Insight on Edible Insect Protein: Extraction, Functional Properties, Allergenicity, Bioactivity, and Applications. Foods.

[25] Li H., Liu Y., Seephua N., Prakitchaiwattana C., Liu R.-X., Zheng J.-S., Siriamornpun S. (2025). Fortification of cricket and silkworm pupae powders to improve nutritional quality and digestibility of rice noodles. Food Chem. X.

[26] Cruz V.A., Vicentini-Polette C.M., Magalhaes D.R., de Oliveira A.L. (2025). Extraction, Characterization, and Use of Edible Insect Oil—A Review. Food Chem..

[27] Andrade R., Martins L.L., Mourato M.P., Lourenço H., Ramos A.C., Roseiro C., Pereira N., Costa G.J., Lucas R., Alvarenga N. (2025). Nutritional and Microbial Quality of Edible Insect Powder from Plant-Based Industrial By-Product and Fish Biowaste Diets. Foods.

[28] Gantner M., Sadowska A., Piotrowska A., Kulik K., Sionek B., Kostyra E. (2024). Wheat Bread Enriched with House Cricket Powder *(Acheta domesticus* L.) as an Alternative Protein Source. Molecules.

[29] Yeerong K., Chantawannakul P., Anuchapreeda S., Wangtueai S., Chaiyana W. (2024). Optimization of Hydrolysis Conditions, Isolation, and Identification of Biologically Active Peptides Derived from *Acheta domesticus* for Antioxidant and Collagenase Inhibition. Antioxidants.

[30] Aiello D., Barbera M., Bongiorno D., Cammarata M., Censi V., Indelicato S., Mazzotti F., Napoli A., Piazzese D., Saiano F. (2023). Edible Insects an Alternative Nutritional Source of Bioactive Compounds: A Review. Molecules.

[31] Brai A., Pasqualini C., Poggialini F., Vagaggini C., Dreassi E. (2025). Insects as Source of Nutraceuticals with Antioxidant, Antihypertensive, and Antidiabetic Properties: Focus on the Species Approved in Europe up to 2024. Foods.

[32] Vehar A., Potocnik D., Strojnik L., Zuliani T., Heath D., Mencin M., Vrhovsek U., Skvorová P., Kourimská L., Kulma M. (2025). Nutritional Composition of Farmed Insects: Impact of Species, Developmental Stage, and Sex. J. Insects Food Feed.

[33] Izadi H., Asadi H., Bemani M. (2025). Chitin: A Comparison between Its Main Sources. Front. Mater..

[34] Nino M.C., Reddivari L., Ferruzzi M.G., Liceaga A.M. (2021). Targeted Phenolic Characterization and Antioxidant Bioactivity of Extracts from Edible *Acheta domesticus*. Foods.

[35] Umebara I., Akutsu K., Kubo M., Iijima A., Sakurai R., Masutomi H., Ishihara K. (2024). Analysis of Fatty Acid Composition and Volatile Profile of Powder from Edible Crickets (*Acheta domesticus*) Reared on Apple By-Products. Foods.

[36] Lu M., Zhu C., Smetana S., Zhao M., Zhang H., Zhang F., Du Y. (2024). Minerals in Edible Insects: A Review of Content and Potential for Sustainable Sourcing. Food Sci. Hum. Wellness.

[37] Mabelebele M., Kolobe S.D., Malematja E., Sebola N.A., Manyelo T.G. (2023). A Comprehensive Review of the Importance of Selected Trace Elements Present in Edible Insects. Biol. Trace Elem. Res..

[38] Malematja E., Sebola N.A., Manyelo T.G., Kolobe S.D., Mabelebele M. (2023). Copping out of Novel Feeds: HOW Climate Change Pledgers and Food Summits Overlooked Insect Protein. Heliyon.

[39] Mwangi M.N., Oonincx D.G.A.B., Hummel M., Utami D.A., Gunawan L., Veenenbos M., Zeder C., Cercamondi C.I., Zimmermann M.B., Van Loon J.J.A. (2022). Absorption of Iron from Edible House Crickets: A Randomized Crossover Stable-Isotope Study in Humans. Am. J. Clin. Nutr..

[40] Cunha N., Andrade V., Macedo A., Ruivo P., Lima G. (2025). Methods of Protein Extraction from House Crickets (*Acheta domesticus*) for Food Purposes. Foods.

[41] Gravel A., Doyen A. (2020). The Use of Edible Insect Proteins in Food: Challenges and Issues Related to Their Functional Properties. Innov. Food Sci. Emerg. Technol..

[42] López-Gámez G., del Pino-García R., López-Bascón M.A., Verardo V. (2024). From Feed to Functionality: Unravelling the Nutritional Composition and Techno-Functional Properties of Insect-Based Ingredients. Food Res. Int..

[43] Mannozzi C., Foligni R., Mozzon M., Aquilanti L., Cesaro C., Isidoro N., Osimani A. (2023). Nonthermal Technologies Affecting Techno-Functional Properties of Edible Insect-Derived Proteins, Lipids, and Chitin: A Literature Review. Innov. Food Sci. Emerg. Technol..

[44] Queiroz L.S., Nogueira Silva N.F., Jessen F., Mohammadifar M.A., Stephani R., Fernandes de Carvalho A., Perrone Í.T., Casanova F. (2023). Edible Insect as an Alternative Protein Source: A Review on the Chemistry and Functionalities of Proteins under Different Processing Methods. Heliyon.

[45] Sadeghi A., Ebrahimi M., Assadpour E., Jafari S.M. (2025). Application of Edible Insects in Bread Enrichment; Emerging Techno-Functional Opportunities and Potential Challenges. Future Foods.

[46] Lin X., Wang F., Lu Y., Wang J., Chen J., Yu Y., Tao X., Xiao Y., Peng Y. (2023). A Review on Edible Insects in China: Nutritional Supply, Environmental Benefits, and Potential Applications. Curr. Res. Food Sci..

[47] De Marchi L., Wangorsch A., Zoccatelli G. (2021). Allergens from Edible Insects: Cross-Reactivity and Effects of Processing. Curr. Allergy Asthma Rep..

[48] de Matos F.M., Rasera G.B., Soares De Castro R.J. (2024). Insects as a Sustainable Source of Emerging Proteins and Their Processing to Obtain Bioactive Compounds: An Updated Review. Sustain. Food Technol..

[49] Page M.J., McKenzie J.E., Bossuyt P.M., Boutron I., Hoffmann T.C., Mulrow C.D., Shamseer L., Tetzlaff J.M., Akl E.A., Brennan S.E. (2021). The PRISMA 2020 Statement: An Updated Guideline for Reporting Systematic Reviews. BMJ.

[50] Vanhecke T.E. (2008). Zotero. J. Med. Libr. Assoc..

[51] Ouzzani M., Hammady H., Fedorowicz Z., Elmagarmid A. (2016). Rayyan—A Web and Mobile App for Systematic Reviews. Syst. Rev..

[52] Rossi S., Parrotta L., Del Duca S., Rosa M.D., Patrignani F., Schluter O., Lanciotti R. (2021). Effect of *Yarrowia Lipolytica* RO25 Cricket-Based Hydrolysates on Sourdough Quality Parameters. LWT.

[53] Patrignani F., Parrotta L., Del Duca S., Vannini L., Camprini L., Dalla Rosa M., Schlüter O., Lanciotti R. (2020). Potential of *Yarrowia Lipolytica* and *Debaryomyces Hansenii* Strains to Produce High Quality Food Ingredients Based on Cricket Powder. LWT.

[54] Rossi S., Parrotta L., Gottardi D., Glicerina V.T., Del Duca S., Rosa M.D., Patrignani F., Schlüter O., Lanciotti R. (2022). Unravelling the Potential of Cricket-Based sHydrolysed Sourdough on the Quality of an Innovative Bakery Product. J. Insects Food Feed.

[55] Bartkiene E., Zokaityte E., Starkute V., Zokaityte G., Kaminskaite A., Mockus E., Klupsaite D., Cernauskas D., Rocha J.M., Özogul F. (2023). Crickets (*Acheta domesticus*) as Wheat Bread Ingredient: Influence on Bread Quality and Safety Characteristics. Foods.

[56] Kowalczewski P.Ł., Gumienna M., Rybicka I., Górna B., Sarbak P., Dziedzic K., Kmiecik D. (2021). Nutritional Value and Biological Activity of Gluten-Free Bread Enriched with Cricket Powder. Molecules.

[57] Nissen L., Samaei S.P., Babini E., Gianotti A. (2020). Gluten Free Sourdough Bread Enriched with Cricket Flour for Protein Fortification: Antioxidant Improvement and Volatilome Characterization. Food Chem..

[58] Galli V., Venturi M., Pini N., Granchi L. (2020). Technological Feature Assessment of Lactic Acid Bacteria Isolated from Cricket Powder’s Spontaneous Fermentation as Potential Starters for Cricket-Wheat Bread Production. Foods.

[59] Cappelli A., Oliva N., Bonaccorsi G., Lorini C., Cini E. (2020). Assessment of the Rheological Properties and Bread Characteristics Obtained by Innovative Protein Sources (*Cicer arietinum*, *Acheta domesticus*, *Tenebrio molitor*): Novel Food or Potential Improvers for Wheat Flour?. LWT.

[60] Belleggia L., Foligni R., Ferrocino I., Biolcati F., Mozzon M., Aquilanti L., Osimani A., Harasym J. (2023). Morphotextural, Microbiological, and Volatile Characterization of Flatbread Containing Cricket (*Acheta domesticus*) Powder and Buckwheat (*Fagopyrum esculentum)* Flour. Eur. Food Res. Technol..

[61] Karwacka K., Łobacz A., Ziajka J., Lis A., Małkowska-Kowalczyk M., Baranowska M. (2024). Use of House Cricket (*Acheta domesticus*) Powder in Yoghurt Products. Foods.

[62] Dridi C., Millette M., Uscanga B.R.A., Salmieri S., Allahdad Z., Lacroix M. (2023). Evaluation of the Nutritional Quality and In Vivo Digestibility of Probiotic Beverages Enriched with Cricket Proteins. Food Bioprocess Technol..

[63] Kittibunchakul S., Whanmek K., Santivarangkna C. (2023). Physicochemical, Microbiological and Nutritional Quality of Fermented Cricket (*Acheta domesticus*) Paste. LWT.

[64] Dhakal M., Kemsawasd V., Whanmek K., Chathiran W., Intawong S., Srichamnong W., Suttisansanee U., Kittibunchakul S. (2025). Physicochemical Characteristics, Volatile Components and Bioactivities of Fermented Seasoning Sauce Produced from Cricket (*Acheta domesticus*) Meal. Future Foods.

[65] Yang J., Chen Y., Zhang L., Zhou S., You L., Song J. (2025). Application of Edible Insects to Food Products: A Review on the Functionality, Bioactivity and Digestibility of Insect Proteins under High-Pressure/Ultrasound Processing. Food Chem..

[66] Roncolini A., Milanović V., Cardinali F., Osimani A., Garofalo C., Sabbatini R., Clementi F., Pasquini M., Mozzon M., Foligni R. (2019). Protein Fortification with Mealworm (*Tenebrio Molitor* L.) Powder: Effect on Textural, Microbiological, Nutritional and Sensory Features of Bread. PLoS ONE.

